# Supplementary material for: Content-rich biological network constructed by mining PubMed abstracts
Source: BMC Bioinformatics. 2004 Oct 8;5:147. doi: 10.1186/1471-2105-5-147 (PMC528731; doi:10.1186/1471-2105-5-147)
Supplement: Additional File 2 — The original results of the above study (non-essential files are deleted to keep the file size under the limit set by BMC bioinformatics). [file 1471-2105-5-147-S2.bz2 › chilibotAdditionalFile2/dip40/364ID8663044E6037/html/FGFR2_FGF3.html]

 


 **FGFR2** and **FGF3** 
  
Found 33 abstracts in PubMed, retrieved 33.  
 

 What does Google say? 
 PDF only 
| .edu only 

---

**Interactive relationship** (e.g. stimulation, inhibition, etc)

**Neutral relationship**- In addition, hindbrain derived  **FGF3**  might regulate otocyst morphogenesis through FGFR 2  [ **FGFR2** ]  IIIb.  Ref: 10934262 J Neurosci, 2000
- BEK  [ **FGFR2** ]  was found significantly correlated with MYC and FLG with HST INT2  [ **FGF3** ]  BLC1.  Ref: 1851551 Oncogene, 1991
- Fibroblast growth factor FGF 3  [ **FGF3** ]  from Xenopus laevis XFGF3 binds with high affinity to FGF receptor  [ **FGFR2** ]  2.  Ref: 7896824 J Biol Chem, 1995
- Since int 2  [ **FGF3** ]  and kgf strongly bind the same receptor, the IIIb isoform of  **FGFR2** , it is possible that their joint expression, one as a transgene, the other as an activated gene, might reinforce the same mitogenic pathway.  Ref: 9000125 Oncogene, 1996
- Using a receptor binding competition assay,  **Fgf3**  was shown to bind with high affinity to the IIIb isoforms of Fgf receptor  [ **FGFR2** ]  FgfR 1 and  **FgfR2**  ID50 = approximately 0.8 nM and with a lower affinity to the IIIc variant of  **FgfR2**  ID50 = approximately 9 nM.  Ref: 7592624 J Biol Chem, 1995
- Keratinocyte growth factor receptor  [ **FGFR2** ]  KGFR  [ **FGFR2** ] , also known as fibroblast growth factor receptor FGFR 2  [ **FGFR2** ]  IIIb, is located in many types of epithelial cells and is activated by four known ligands FGF 1, FGF 3  [ **FGF3** ] , FGF 7 also known as KGF and FGF 10 that are predominantly synthesized by mesenchymal cells.  Ref: 12608893 Pathol Int, 2003
- Expression of the cysteine rich fibroblast growth factor FGF receptor  [ **FGFR2** ]  CFR in COS 1 cells strongly inhibits the secretion of co expressed  **FGF3** .  Ref: 10748074 J Biol Chem, 2000

**Non-interactive relationship** (e.g. studied together, co-existance, homology, etc.)

- The truncated  **FGFR2** , the high affinity FGFR for  **FGF3** , is significantly more efficient at reverting the transformed phenotype as the IIIc isoform, reaffirming the notion that the affinity of the ligand to the DN  **FGFR2**  isoform determines the effect.  Ref: 12084721 J Biol Chem, 2002
- Since the same tumors were previously analysed for the amplification of MYC, ERBB2 and HST INT2  [ **FGF3** ]  BCL1 possible associations with BEK  [ **FGFR2** ]  and FLG amplifications were looked for.  Ref: 1851551 Oncogene, 1991
- Since FGF 1 and FGF 3  [ **FGF3** ]  can both activate one FGF receptor  [ **FGFR2** ]  isoform  **FGFR2**  IIIb that is not activated by FGF 2, these results suggest that activation of  **FGFR2**  IIIb is sufficient to induce fiber cell differentiation throughout the lens epithelium in vivo.  Ref: 9640329 Dev Biol, 1998
- In later development,  **FGF3**  or FGF10 acting on FGF receptor  [ **FGFR2** ]  2b is likely to be involved in development of the walls of the cochlear spaces, while FGF receptor  [ **FGFR2** ]  3 is involved in differentiation of the pillar cells of the organ of Corti.  Ref: 11914524 Audiol Neurootol< MedlineTA>Audiol Neurootol, 1998
- The expression of  **FGF3**  together with the FGF receptors FGFR1,  **FGFR2** , and FGFR3, was detected in the mouse tumors by Northern blot analysis.  Ref: 9645456 J Cancer Res Clin Oncol, 1998
